# Calciphylaxis in a nondialysis patient treated with sodium thiosulfate and high dose of oxygen 

**DOI:** 10.5414/CNCS108959

**Published:** 2017-07-06

**Authors:** Anawin Sanguankeo, Natanong Thamcharoen, Sikarin Upala

**Affiliations:** 1 Department of Internal Medicine, Bassett Medical Center and Columbia University College of Physicians and Surgeons, Cooperstown, NY, USA, and; 2 Department of Preventive and Social Medicine, Faculty of Medicine Siriraj Hospital, Mahidol University, Thailand

**Keywords:** calciphylaxis, nondialysis, sodium thiosulfate

## Abstract

Background: Calciphylaxis in a nondialysis patient is a rare condition and is characterized by calcific deposition in tissue. We present a case of calciphylaxis in a nondialysis patient who was diagnosed by clinical presentation and skin biopsy and was treated with sodium thiosulfate with improvement of skin lesions. Case: A 43-year-old female with type 2 diabetes and atrial fibrillation taking oral anticoagulation medication presented with reddish drainage from the right buttock. On physical examination, a large perirectal abscess overlying necrosis was found. She also developed acute kidney injury with creatinine of 3.7 mg/dL at peak from 0.8 mg/dL at baseline. She received antibiotics intravenously and wound debridement. During hospitalization, she developed areas of numerous painful erythematous lesions with central dusky necrosis on bilateral lower extremities. Punch biopsy was done, which initially revealed small-vessel vasculitis. However, those lesions did not respond to steroid therapy. A second biopsy was done showing extensive fat necrosis and medial calcification of vessel walls consistent with calciphylaxis. She was treated with high-flow oxygen and sodium thiosulfate intralesionally and intravenously for 6 months. The lesions remarkably reduced in size and were less painful on follow-up. Conclusion: High-dose oxygen and sodium thiosulfate could potentially be effective treatments for calciphylaxis in nondialysis patients.

## Introduction 

Calciphylaxis or calcific uremic arteriolopathy (CUA) is a rare complication in end-stage renal disease (ESRD) patients, particularly in dialysis-dependent patients [1]. Presentation for this condition mostly occurs in high-adipose tissue areas and is characterized by painful ischemic necrotic plaque/nodules that can progress to ischemic ulcers and eventually become complicated by infection. Although the pathophysiology of calciphylaxis is still poorly understood, it is believed that calciphylaxis is associated with disorders of calcium, phosphate, and parathyroid hormone homeostasis in end-stage kidney disease [2]. Calciphylaxis in a non-dialysis patient is a rare form of this condition in the absence of end-stage kidney disease and renal transplantation. We present a case of calciphylaxis in a nondialysis patient who was resuscitated and treated successfully with improvement of the lesions. 

## Case presentation 

A 43-year-old female was admitted for perirectal abscess. Her comorbid conditions included hypertension, obstructive sleep apnea, type 2 diabetes, atrial fibrillation, morbid obesity, and rheumatoid arthritis (treated with chronic steroid therapy). The patient’s current presentation began with 1 month of reddish drainage from the right buttock. She reported no trauma, fever, or other systemic symptoms. On physical examination, she had a large perirectal abscess sized 12.24 × 8.56 cm with overlying necrosis and extensive erythema up to the lower back with discharge of brown-red material. Her creatinine was 3.7 mg/dL, which increased from her baseline of 0.8 mg/dL. She was started on intravenous cefoxitin and clindamycin. The findings revealed 10 – 12 cm of necrosis and left perirectal abscess involving the ischiorectal fossa but not extending to the rectum. Postoperatively, she was transferred to the ICU as she was hypotensive, requiring vasopressor infusion. The patient was taken to the operating room multiple times for debridement and split-thickness skin grafts from her left posterior thigh. Her condition continued to improve, and she was able to be moved out of the ICU. Her creatinine came back to 1 mg/dL. 

**Figure 1.**
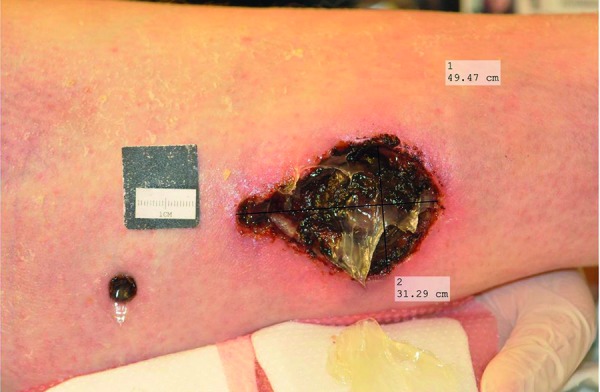
Necrotic lesion on right lower extremity.

On hospital day 17, she developed areas of numerous severely sharp, painful erythematous lesions with central dusky necrosis and subsequently ulcerated lesions ranging in size from 1 to 5 cm on the bilateral lower extremities (Figure 1). Punch biopsy from these lesions was done. The final pathologic diagnosis was small-vessel vasculitis. It was thought that the lesions were pyoderma gangrenosum related to her underlying rheumatoid arthritis. Her steroid dose was increased to prednisone 30 mg daily for lesion treatment. The labs were sent off for the genetic hypercoagulable panel, including protein C, protein S, cryofibrinogen, reptilase time, DRVVT screen ratio, prothrombin G20210A mutation, lupus anticoagulant, and antithrombin III, which were all negative. The patient was discharged to a short-term rehab facility as her condition had improved. Three days later, she was readmitted again for sepsis syndrome due to infected ulcers on the bilateral lower extremities. She developed fever up to 38.2 °C and was hypotensive. Broad-spectrum antibiotic, vancomycin, and piperacillin-tazobactam were initiated again. Since the patient had numerous non-healing bilateral lesions on her lower extremities, which did not respond to steroids, skin biopsy of the same lesion was performed again and sent to a pathology laboratory in a tertiary facility. Final pathology showed extensive fat necrosis and lymphohistiocytic inflammatory cell infiltrate. Medial calcification of vessel walls was seen focally, and some pockets of neutrophils were visible. These findings were consistent with calciphylaxis. On review of the record, the patient also had secondary hyperparathyroidism due to vitamin D deficiency (Table 1). Her calcium and phosphorus were in the normal range. The intact parathyroid hormone (PTH) was mildly elevated at 88 pg/mL (14 – 72 pg/mL). Total vitamin D level was 11 ng/mL (31 – 100 ng/mL). Consequently, she was started on treatment with 15 L of oxygen by facemask for 2 hours a day, sodium thiosulfate 25 g intravenously 5 days per week, and sodium thiosulfate intralesional injections every 7 days. Daily prednisone 30 mg was maintained for rheumatoid arthritis treatment. Oxycodone, oral morphine, gabapentin, and acetaminophen were used to control the ulcer pain. Vitamin D level was repleted with cholecalciferol 1,000 µg per day. 

**Figure 2.**
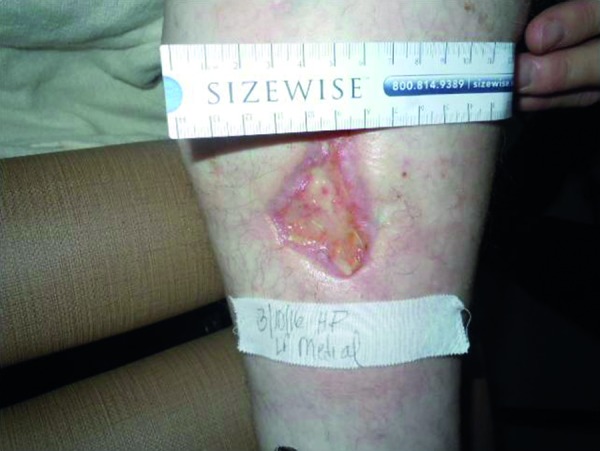
Healed lesion after 6 months.

On 6-month follow-up, the ulcerated lesions had satisfactorily improved (Figure 2). Two out of four ulcer regions on her lower extremities were healed. The two remaining were in the process of healing. She has made good improvement in physical function. 

## Discussion 

As in our patient, although it is rare, calciphylaxis can occur in patients without ESRD requiring dialysis. Sepsis was the leading cause of death, and overall mortality rate was 52% for the nondialysis patients with calciphylaxis [2]. Clinical features of calciphylaxis skin lesions range from palpable firm calcified subcutaneous tissue, livedo reticularis, reticulate purpura, and violaceous plaque related to severe pain at the lesions [3]. Definite diagnosis can be obtained by lesion margin biopsy demonstrating vascular calcification, small artery proliferation leading to microthrombosis and tissue ischemia as seen in our patient [4]. The pathogenesis of calciphylaxis is still poorly understood. In uremic calciphylaxis, it is believed that pathogenesis is related to an imbalance of calcium-phosphorous metabolism leading to calcification of vascular structure. However, dysregulation of calcium and phosphorus alone cannot be applied as a cause of calciphylaxis in a nondialysis patient. Hypercoagulability has also been suggested to play a significant role in this condition [5]. In a systematic review, primary hyperparathyroidism, connective tissue diseases, alcoholic liver disease, malignancies, diabetes, chemotherapy-induced protein C and S deficiency, Crohn disease, POEMS syndrome, vitamin D deficiency, weight loss, corticosteroid use, warfarin use, and osteomalacia treated with nadroparin calcium were reported to be associated with calciphylaxis in nondialysis patients [2]. Our patient’s risk factors for calciphylaxis included secondary hyperparathyroidism due to vitamin D deficiency, diabetes, female sex, obesity, rheumatoid arthritis, and warfarin use. Differential diagnoses for calciphylaxis in nondialysis patients are peripheral vascular disease, vasculitic diseases, antiphospholipid syndrome, heparin-induced skin necrosis, purpura fulminans, and warfarin-induced skin necrosis [6]. The clinician should rule out all possibilities that can mimic this condition and look for the cause of calciphylaxis in a nondialysis patient by evaluating renal function, serum calcium, phosphorus, parathyroid hormone, vitamin D, liver enzymes, coagulation and hypercoagulation status, autoimmune disease, and malignancy. This patient did not have hypercoagulable syndrome. 

At present, there is no standard treatment of calciphylaxis due to limited clinical studies and cases. Treatment for calciphylaxis patients requires a multidisciplinary approach. Intravenous sodium thiosulfate and removing potential triggers are the main medical treatment for calciphylaxis. Successful treatment with intralesional sodium thiosulfate injection was also reported [7]. Supportive treatment, such as pain control and wound care, are also very important as patients mostly suffer from severe pain complicated by wound infection. Various modalities of wound management have been reported with effective results, including hyperbaric oxygen therapy and sterile maggot therapy [5, 6]. In addition to typical thiosulfate infusion, we planned to initiate treatment with hyperbaric oxygen, but the patient had financial issues. High-dose oxygen therapy was applied for substitution with good effect. There has been no evidence of high-dose oxygen therapy for calciphylaxis in a nondialysis patient before; this method is more convenient than hyperbaric oxygen. Therefore, high-dose oxygen could potentially be applied for calciphylaxis in substitution of hyperbaric treatment. 

## Conflict of interest 

All authors declare no conflict of interest. 

**Table 1.** Markers of secondary hyperparathyroidism.

	Initial presentation	Follow-up at 3 months
Total calcium (8.4 – 10.2 mg/dL)	9.3	9.2
Ionized calcium (4.2 – 5.5 mg/dL)	5.5	3.4
Phosphorus (2.5 – 4.9 mg/dL)	3.9	3.2
Alkaline phosphatase (38 – 125 U/L)	159	229
PTH (14 – 72 pg/mL)	88	133
25-hydroxyvitamin D (20 – 50 ng/mL)	11	26
